# Comparison of Oxide Scale Morphology on FeAl-Based Alloy After Long-Term Oxidation in Air and Water Vapor at 700 °C

**DOI:** 10.3390/ma19071459

**Published:** 2026-04-05

**Authors:** Janusz Cebulski, Dorota Pasek, Maria Sozańska, Magdalena Popczyk, Jadwiga Gabor, Andrzej Swinarew

**Affiliations:** 1 Department of Materials Technology, Faculty of Materials Engineering and Industrial Digitalization, Silesian University of Technology, Krasińskiego 8, 40-019 Katowice, Poland; maria.sozanska@polsl.pl; 2 Promobil s.c., Kopernika 12, 40-064 Katowice, Poland; dorota.pasek@promobil.pl; 3 Faculty of Science and Technology, University of Silesia in Katowice, 75 Pułku Piechoty 1A, 41-500 Chorzów, Poland; 4 Department of Training and Nutrition in Sports, The Jerzy Kukuczka Academy of Physical Education, Mikołowska 72A, 40-065 Katowice, Poland

**Keywords:** oxidation, oxide layer, SEM, Al_2_O_3_, FeAl

## Abstract

**Highlights:**

**The main findings?**

- An ultrathin α-Al_2_O_3_-based oxide scale (~300 nm) forms after 2000 h at 700 °C.
- The oxide layer exhibits locally heterogeneous morphology (whisker-like and fine-crystalline features).
- The EDS signal is significantly influenced by substrate contribution even at low accelerating voltages.

**The implications of the main findings?**

- The reliability of SEM-EDS analysis for ultrathin oxide scales is strongly dependent on accelerating voltage.
- Monte Carlo simulations are essential for correct interpretation of information depth in thin-layer systems.
- Accurate characterization requires a combined use of SEM-EDS/WDS, XRD, and EBSD techniques.

**Abstract:**

The present study investigates the morphology, chemical composition, and phase constitution of oxide scales formed on the Fe40Al5Cr0.2TiB intermetallic alloy after long-term oxidation at 700 °C for 2000 h in air and water vapor environments. The results demonstrate the formation of an extremely thin oxide scale (≈300 nm), composed predominantly of α-Al_2_O_3_, which provides effective protection against further oxidation. The oxide layer exhibits locally heterogeneous morphology, including whisker-like structures and fine crystallites. Due to the very limited thickness of the oxide scale, significant challenges arise in the interpretation of microanalytical data. It is shown that the accelerating voltage strongly influences the effective information depth in SEM-EDS analysis, leading to a substantial contribution from the substrate even at low voltages. Monte Carlo simulations were used to support the interpretation of electron–matter interactions and to explain the observed discrepancies in chemical analysis. The study demonstrates that reliable characterization of ultrathin oxide scales requires careful optimization of SEM parameters and the combined use of complementary techniques, including EDS/WDS, XRD, and EBSD. The findings highlight the importance of methodological considerations in the analysis of thin oxide layers and provide guidance for the correct interpretation of experimental data in similar systems.

## 1. Introduction

Studies of the morphology, chemical composition, and phase constitution of oxidation products formed on alloys based on Fe–Al intermetallic phases require the application of advanced research methods and techniques. Although oxidation can be considered one of the simplest chemical reactions, its mechanisms are often complex and difficult to analyse in detail.

The allotropic forms and morphology of Al_2_O_3_ oxides formed on multi-component alloys depend on the chemical composition of the material and the thermodynamic conditions of the process. The structure and growth mechanism of the protective Al_2_O_3_ scale on heat-resistant alloys strongly depend on the alloying elements present in the matrix [1,2,3,4]. Aluminum oxides occur in various polymorphic forms depending on the oxidation temperature, but α-Al_2_O_3_ is the only thermodynamically stable phase [5,6]. Other forms (γ, δ, and θ) are transitional and provide less effective protection. These transition oxides are typically formed at lower temperatures and transform into α-Al_2_O_3_ depending on time, temperature, and alloy composition [7,8].

In the temperature range of 800–950 °C, the initial stages of oxidation of FeAl-based alloys are associated with the formation of metastable alumina phases (θ, γ, δ), which gradually transform into a protective α-Al_2_O_3_ layer. One of the proposed explanations for this behavior involves epitaxial effects [9,10]. Most studies available in the literature focus on oxidation resistance in the temperature range of 800–1100 °C.

In situ XRD studies reported in [11] for FeAl-based alloys showed that oxidation begins at relatively low temperatures, with γ-Fe_2_O_3_ formation at 400 °C and the appearance of FeAl_2_O_4_ spinel at 500 °C. At 700 °C, a decrease in γ-Fe_2_O_3_ peaks and an increase in FeAl reflections were observed, while Al_2_O_3_ was first detected at temperatures above 800 °C. These results suggest that 700 °C may correspond to the initial stage of alumina formation. However, due to the very limited thickness of the oxide layer at this temperature, advanced and carefully selected characterization methods are required for reliable identification of oxidation products.

At the same time, the characterization of such thin oxide scales poses significant methodological challenges. The analytical signal obtained from techniques such as SEM-EDS or XRD may be strongly affected by the contribution from the underlying substrate, which can lead to misinterpretation of the chemical and phase composition. Therefore, proper consideration of the information depth and careful selection of experimental parameters are essential for reliable analysis.

Previous studies by the authors have addressed various aspects of FeAl-based alloys, including the influence of processing conditions on material properties [12], general oxidation behaviour in a wider temperature range [13], and the structure and corrosion resistance of Fe40Al5Cr0.2TiB alloy [14]. In addition, the potential applications and heat resistance of FeAl intermetallic alloys have been discussed in [15].

However, these studies did not focus on the formation and characterization of ultrathin oxide scales at relatively low temperatures, nor on the methodological limitations associated with their analysis. The present work extends these investigations by addressing long-term oxidation at 700 °C and by analysing the reliability and limitations of commonly used characterization techniques in the case of very thin oxide layers.

The aim of the present study is to evaluate the morphology, chemical composition, and phase constitution of oxidation products formed on the Fe40Al5Cr0.2TiB alloy after long-term oxidation at 700 °C for 2000 h.

In contrast to previous studies on FeAl- and FeCrAl-based alloys, which mainly focus on higher oxidation temperatures and thicker oxide scales, the present work addresses the formation and characterization of an exceptionally thin oxide layer (on the order of a few hundred nanometers) formed at a relatively low temperature.

Particular attention is given to the methodological aspects of oxide scale characterization, especially the limitations of SEM-based microanalysis in the case of very thin layers. The effect of accelerating voltage on the effective information depth and the resulting contribution from the substrate is systematically analyzed and supported by Monte Carlo simulations of electron–matter interactions.

The study demonstrates that reliable interpretation of chemical and phase composition for ultrathin oxide scales requires careful optimization of SEM operating conditions and cross-validation using complementary techniques such as EDS/WDS, XRD, and EBSD.

## 2. Materials and Methods

The tests were carried out on samples cut from the cast Fe40Al5Cr0.2TiB alloy (the detailed chemical composition given in Table 1). The melts were carried out in the VSG-2 vacuum induction furnace by Balzers (Balzers, Lichtenstein). Oxidation tests were carried out at 700 °C for 2000 h under two different environments: atmospheric air and water vapor.

In the case of air exposure, the samples were oxidized in a chamber furnace under laboratory atmospheric air (pO_2_ ≈ 0.21 atm).

For oxidation in water vapor, a flow-through system with a tube furnace was used. Water vapor was generated from distilled water delivered by a pump to a steam generator at a rate of approximately 200 mL/h and then passed through a superheater before entering the furnace chamber. Under the applied conditions (700 °C), the atmosphere consisted of superheated steam (H_2_O gas), providing continuous flow through the reaction zone. The applied conditions ensured a stable and continuous supply of the oxidizing medium during long-term exposure.

The oxide layer was grown on small platelets (Ø 6 mm) cut from the ingot. In order to determine the thickness of the coating formed after the corrosion tests, the oxidation samples were nickel-plated to prevent corrosion products from falling off during preparation. Observations were carried out using the Hitachi S-3400N (Hitachi High-Tech Corp., Tokyo, Japan) and Hitachi S-4200 (SEM) (Hitachi High-Tech Corp., Tokyo, Japan) scanning electron microscope using a secondary electron (SE) and backscattered electron (BSE) detector. Chemical composition tests of oxidation products on solid samples were carried out using an energy dispersive X-ray spectrometer (EDS) and a wavelength dispersion spectrometer (WDS) by Thermo Noran (System Six) (Waltham, MA, USA) at a voltage accelerating the electron beam of 5, 10, and 15 keV, coupled with a microscope Hitachi S-3400N (Hitachi High-Tech Corp., Tokyo, Japan). Monte Carlo simulations of electron scattering for FeAl alloy and Al_2_O_3_ oxide were carried out in the Single Scattering Monte Carlo Simulation program (CASINO electron simulation program version 3.3.0.4). The Monte Carlo method maps the course of electron scattering in the material by digitally simulating a certain number of electron trajectories and is the most accurate description of the interaction of electrons with the material [16,17,18,19,20,21].

For the purpose of this study, simulations were performed for 10,000 electron trajectories. Qualitative phase analysis of the oxides formed on the surface of the oxidized samples from the Fe40Al5Cr0.2TiB alloy was performed using a JEOL JDX-7S diffractometer (JEOL Ltd., Tokyo, Japan). The radiation source was a lamp with a copper anode.

**Figure 1:**
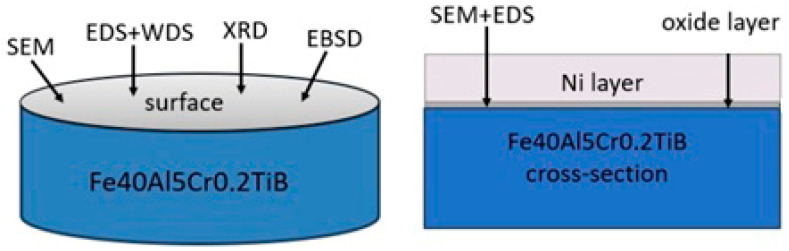
The methodology of the observation of the samples after oxidation.

Backscattered electron diffraction was performed using the EBSD INCA HKL detector and the Nordlys II (Channel 5) analysis system (Hobro, Denmark) equipped with the Hitachi S-3400N microscope (Hitachi High-Tech Corp., Tokyo, Japan). The inclination of the analyzed surface (sample) in relation to the electron-optical axis was 70°. The methodology of the observation of the samples after oxidation is shown in Figure 1.

## 3. Results

### 3.1. Morphology and Thickness of Oxidation Products (SEM)

**Figure 2:**
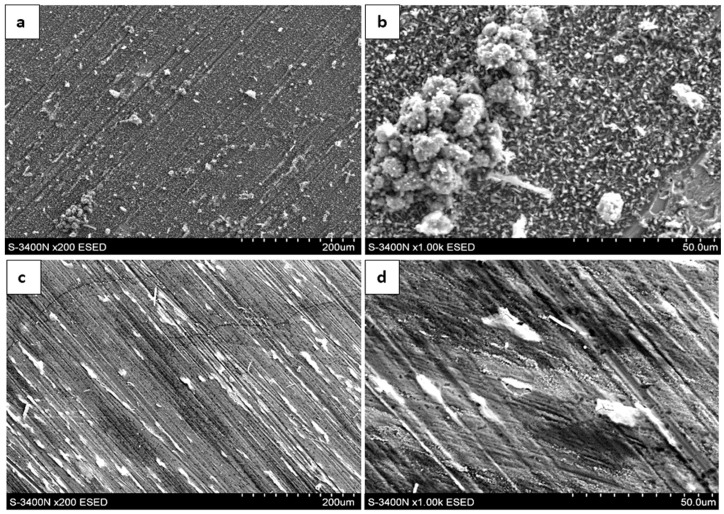
Surface topography after oxidation of the Fe40Al5Cr0.2TiB alloy at 700 °C during 2000 h (SEM): (**a**,**b**)—in steam; (**c**,**d**)—in air.

**Figure 3:**
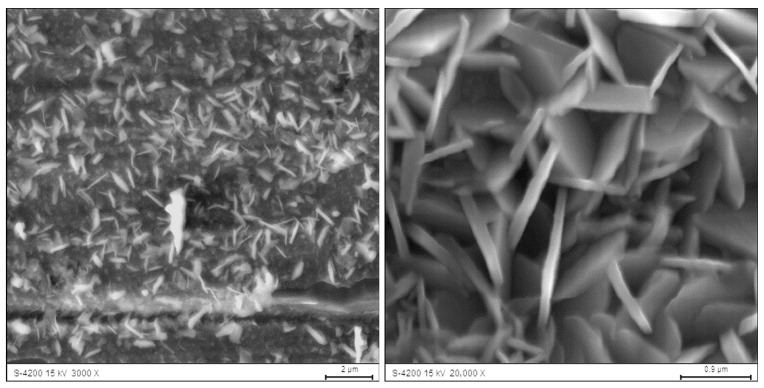
Morphology of oxides formed after the oxidation in air of the Fe40Al5Cr0.2TiB alloy at 700 °C during 2000 h (SEM).

SEM observations revealed that, despite the long oxidation exposure (2000 h at 700 °C), only a small amount of oxidation products is present on the surface, and the oxidation is strongly heterogeneous. In steam, the oxidation products occur mainly as locally developed globular forms accompanied by numerous fine crystallites (Figure 2). In contrast, oxidation in air results in the formation of pronounced needle-/whisker-like features with additional fine surface crystals (Figure 3), indicating locally varying growth conditions and kinetics in the examined environments.

**Figure 4:**
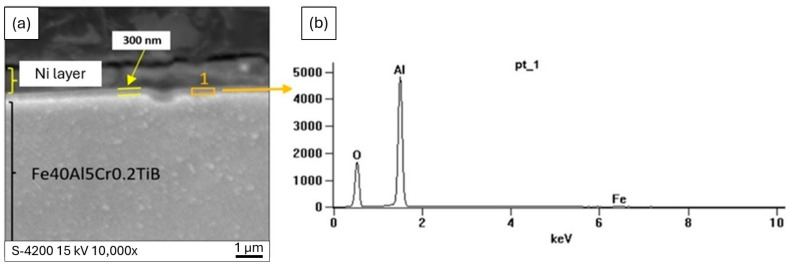
(**a**) Cross-section of Fe40Al5Cr0.2TiB alloy after oxidation in air (SEM), (**b**) EDs X-ray spectrum at the location marked (pt 1) in Figure 4a.

The thickness of the oxide layer was measured on the polished sections of the oxidized platelets using SEM, as shown in Figure 4a,b. A top layer is formed by nickel deposited to protect the oxides from crumbling out during polishing. The microanalysis of the chemical composition in this area confirmed that it is an aluminium oxide (Figure 4b). Based on SEM observations of cross-sections, the oxide scale thickness was estimated to be on the order of a few hundred nanometers (∽300 ± 50 nm in the analyzed areas).

Given the scale thickness and the local character of the measurements, the reported value should be treated as an approximate thickness for the investigated areas; nevertheless, it clearly confirms that the oxide scale remains very thin even after prolonged oxidation under the studied conditions.

### 3.2. Chemical Composition of Oxidation Products (EDS, WDS)

**Figure 5:**
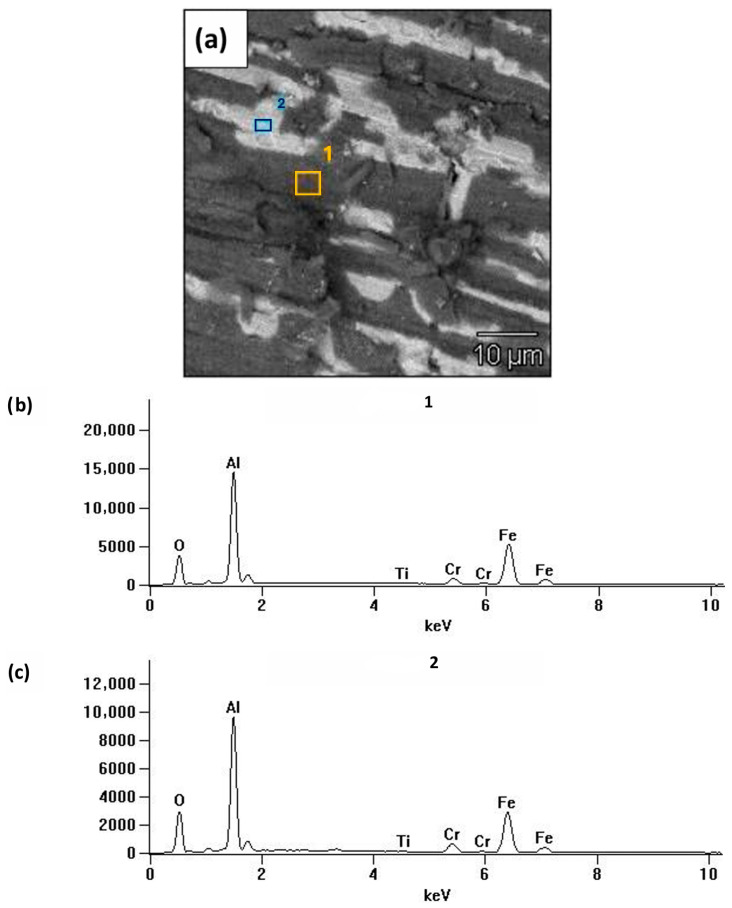
Analysis of the chemical composition of the Fe40Al5Cr0.2TiB alloy after oxidation during 2000 h. (**a**) SEM image of the surface, (**b**,**c**) EDS spectra obtained at the areas marked in Figure 5a.

The qualitative chemical composition of the oxide layer formed on the surface of the samples after oxidation was determined using EDS attachment. The results of the tests are presented in Figure 5. It was found that on the surface of the Fe40Al5Cr0.2TiB alloy after oxidation, a thin layer of scales containing mainly aluminum and oxygen was formed. Apart from oxygen and aluminum, the presence of other elements such as iron, chromium, and titanium was also identified.

**Figure 6:**
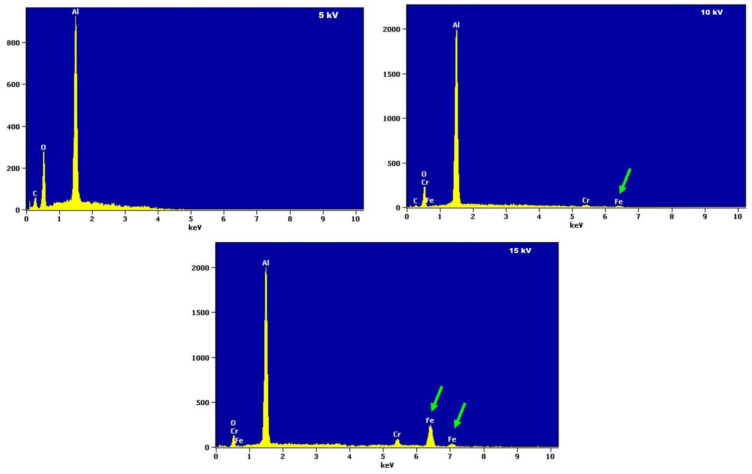
Comparison of the EDS spectra obtained for variable accelerating voltages (5, 10, and 15 kV).

**Figure 7:**
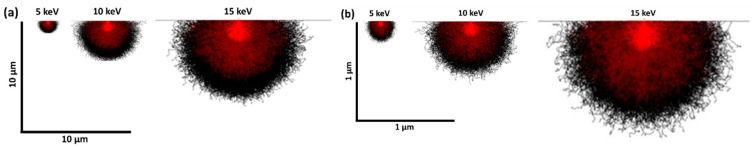
Monte Carlo simulation results for Al_2_O_3_ (**a**) and Fe40Al (**b**).

The obtained result of the EDS analysis carries the signal coming from the substrate. This fact is confirmed by the measurements of the chemical composition analysis (EDS) at 15 kV accelerating voltage (Figure 6). It has been shown that at the accelerating voltage of 5 kV, the identified elements most probably come from the layer of oxidation products (mainly the presence of oxygen and aluminum was found), while with an increase in the accelerating voltage the iron and chromium may show up (Figure 6 and Figure 7).

These results were also confirmed by an additional Monte Carlo simulation experiment, where the excitation area was determined depending on the accelerating voltage of the electron beam for the voltages of 5, 10, 15 kV in the case of FeAl alloy and aluminum oxide (Figure 7).

The simulation performed for Al_2_O_3_ reveals (Figure 7a) that for the accelerating voltage of 5 kV the maximum depth of electron penetration was about 1 µm, for the voltage of 10 kV—about 3 µm, and for the voltage of 15 kV—about 7 µm. Therefore, the effective X-ray generation depth increases markedly with accelerating voltage. In the case of a thin oxide scale, this implies that at 5 kV the recorded EDS signal is dominated by the oxide layer, whereas at 10 kV and 15 kV a significant contribution from the substrate is expected, which affects the apparent elemental composition. At higher accelerating voltage values, the elements in the substrate will also be excited.

If a sufficiently large number of possible electron trajectories is generated by the Monte Carlo simulation method, it is possible to calculate the expected (average) values of the parameters describing the behavior of electrons in the crystal (penetration depth, electron energy at a certain depth, number of scatterings of a specific type). The depth of penetration of primary electrons depends on their energy and the density of the tested material.

**Figure 8:**
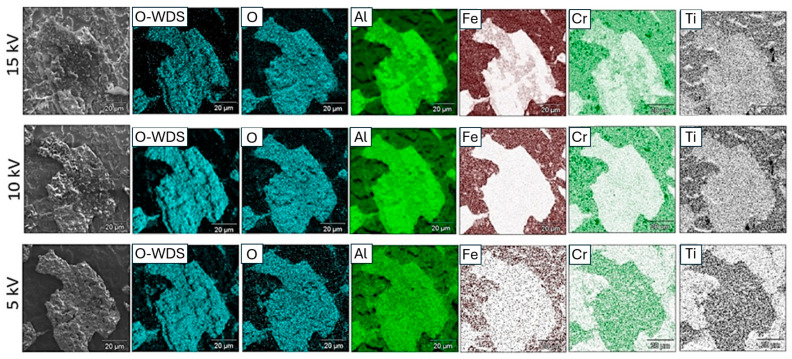
SEM images of surface morphology and accompanying maps of relative concentrations of elements on the sample surface detected by WDS at 5, 10, and 15 kV.

Monte Carlo simulations for a sample of Fe40Al5Cr0.2TiB alloy coated with corrosion products indicate the achievable area of X-ray emission depending on the value of the beam accelerating voltage. This fact is confirmed by the results of the study of the relative concentration of elements on the surface (the so-called mapping—Figure 8). Oxygen analysis was performed using two methods: energy dispersion spectrometry (EDS) and wavelength dispersion spectrometry (WDS). It was found that reducing the accelerating voltage improves oxygen detectability by limiting the interaction volume and reducing substrate contribution. In point EDS spectra (Figure 6), the use of 5 kV resulted in a clearer oxygen peak and a reduced influence of the substrate.

However, in the case of elemental mapping (Figure 8), the signal obtained at 5 kV was insufficient to provide reliable information on the relative distribution of elements across the surface. Under these conditions, the use of 10 kV provided improved signal intensity and better mapping quality, allowing for a more meaningful interpretation of the spatial distribution of oxygen and aluminum.

Therefore, the optimal accelerating voltage depends on the type of analysis: 5 kV is preferable for minimizing substrate contribution in point analysis, whereas 10 kV provides a better compromise for elemental mapping. It is related to the reduction in the critical oxygen excitation energy (overvoltage) multiplication factor with the use of lower accelerating voltage, which improves the efficiency of generating the characteristic X-ray radiation [22,23]. Further reduction in the electron beam accelerating voltage (up to 5 kV) did not result in a noticeable improvement in the image quality obtained in the imaging of the relative concentration of elements on the surface, both for EDS and WDS. The presence of only aluminum and oxygen in areas with different morphology on the surface confirms that these are most likely oxides formed as a result of oxidation.

### 3.3. Phase Composition of Oxidation Products (XRD, EBSD)

**Figure 9:**
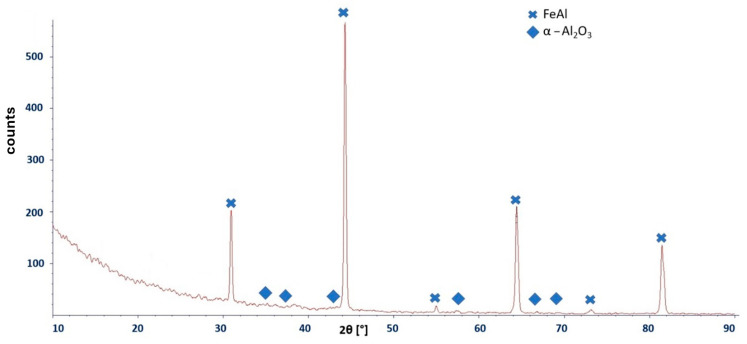
X-ray diffractogram of oxidation products formed on the surface of the Fe40Al5Cr0.2TiB alloy.

The X- Phase identification of the oxidation products formed on the Fe40Al5Cr0.2TiB alloy after oxidation at 700 °C for 2000 h was performed using X-ray diffraction (XRD) and electron backscatter diffraction (EBSD). The XRD results indicate the presence of α-Al_2_O_3_ (Figure 9). At the same time, reflections originating from the FeAl intermetallic substrate are also observed, which is attributed to the limited thickness of the oxide layer and the corresponding penetration depth of the incident X-ray beam. Due to the very limited thickness of the oxide scale, the XRD signal is influenced by the FeAl substrate, as evidenced by the presence of substrate reflections.

**Figure 10:**
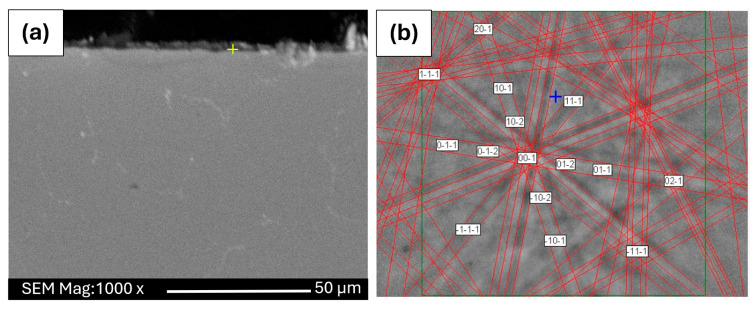
(**a**)—cross-section of the Fe40Al5Cr0.2TiB alloy after oxidation during 2000 h; (**b**)—EBSD pattern recorded at point indicated in Figure 10a, identified as α—Al_2_O_3_ phase.

To verify the oxide phase locally and to reduce ambiguity related to the substrate contribution in XRD, EBSD measurements were carried out on selected surface areas. The EBSD analysis confirms that the crystalline oxidation products correspond to α-Al_2_O_3_ (Figure 10), providing independent support for the XRD-based phase identification.

From the methodological perspective, the agreement between XRD (a predominantly volumetric technique) and EBSD (a local diffraction method) is particularly important for very thin oxidation products, where the diffraction signal may be influenced by the underlying alloy. The identification of α-Al_2_O_3_ as the dominant crystalline oxide is consistent with the observation of only a limited amount of oxidation products after prolonged exposure, and it supports the interpretation that oxidation proceeds with reduced kinetics typical of protective alumina-forming systems. Moreover, the results underline that, under the investigated conditions, phase analysis based solely on a single technique may be biased by information-depth effects, and cross-validation by complementary diffraction methods is required for robust conclusions. Therefore, EBSD analysis was used as a complementary local technique to confirm the phase composition directly within the oxide layer.

## 4. Discussion and Conclusions

The presented work was aimed at the characterisation of the morphology, chemical, and phase composition formed after the oxidation of the alloy Fe40Al5Cr0.2TiB at a temperature of 700 °C after 2000 h.

The morphology of the oxide layer after oxidation in air, as observed by scanning electron microscopy, consisted of areas in the form of coniferous whiskers and crystals growing upward from the substrate. Their formation and growth depend on the local oxidation conditions, as evidenced by their inhomogeneous nature. The applied research methodology made it possible to obtain more information on the morphology of the alumina scales forming on such materials. The obtained results are consistent with the results published in other works on this subject [8,24,25,26,27]. The morphology of Fe40Al5Cr0.2TiB aluminum oxide in the form of thin needles on the surface of the alloy was also demonstrated in the oxidation studies conducted by Nowak K. and Kupka M. [2], however, this form was visible only during oxidation at a higher temperature of 800 °C for 200 h. The coniferous form of oxides on the surface of the alloy was also observed in [28] during oxidation at temperatures of 750 °C and 950 °C, but the process was carried out for 550 h. Similar results concerning oxide morphology were published by Reszka et al. in [29], but the research was carried out on the FeCrAl alloy. The authors indicated that on the surface of the tested material, after the oxidation process, there are oxides of complex morphology, i.e., in the form of fine needles between which the oxide grows in the form of lumps, and the tests were carried out at a temperature of 850 °C.

Determining the chemical composition of oxidation products on the surface of the Fe40Al5Cr0.2TiB alloy by means of energy dispersive spectroscopy (EDS) was limited by the small layer thickness. The difficulty in identifying the elements included in the oxidation products was due to the fact that during the interaction of the electron beam with the surface, the area of excitation of the characteristic X-ray radiation comprised not only the oxidized layer, but also the base material. In such a case, the analysis of the X-ray spectrum prevents an unequivocal assessment of the chemical composition of the analyzed areas. This effect can be reduced by changing the accelerating voltage of the electron beam. By means of X-ray microanalysis of the chemical composition with EDS, it was found that only oxygen and aluminum are present in the oxidized layer. These results were independently confirmed by WDS measurements.

Similar difficulties occurred in the case of X-ray phase analysis (XRD) of oxidation products on the surface. Again, the obtained signal from oxidation products was mixed with that of the substrate. Therefore, the results of the analysis included mainly the base material, and only oxidation products (aluminum oxide) were a small fraction of the spectra. The presence of aluminum oxides was confirmed by XRD analysis by other researchers, but at a higher temperature (approx. 1000 °C) [30,31,32]. The methods and research techniques described there did not allow for obtaining the full characteristics of the oxidized layer to the extent that it was possible to understand its structure and explain the phenomena related to the formation of oxidation products. This is mainly due to the dependence between the resolutions of these test methods and the amount of corrosion products formed after oxidation in both air and water vapor.

Therefore, the phase identification required combining complementary diffraction techniques. The presence of aluminum oxide was confirmed by XRD, and the result was independently verified by EBSD analysis, which allowed local identification of the α-Al_2_O_3_ phase in the near-surface region. At equilibrium conditions, the formation of α-Al_2_O_3_ takes place at a temperature of about 1000 °C [24,25,33]. However, based on research conducted in [26,27], it was found that it can also be formed at temperatures of 700 °C on FeCrAl alloys oxidized in air, which is closer to our findings. Detailed identification of the oxidation products of the Fe40Al5Cr0.2TiB alloy at a temperature of 700 °C after 2000 h required not only the use of advanced methods of material characterization, but also solving methodological problems, in particular related to:

1. Preparation of samples for testing by electron microscopy (securing the oxide against falling off by covering it with an added thin nickel layer).
2. The use of a correctly, precisely selected value of the accelerating voltage in examining the chemical composition by X-ray microanalysis (EDS, WDS), which is necessary due to the small thickness of the oxide layer (as a result, the unwanted signal coming from the substrate is diminished.
3. Cross-validation of phase identification by combining a volumetric diffraction method (XRD) with local diffraction analysis (EBSD) when the oxide-related signal is weak due to a limited amount of corrosion products formed after oxidation.
4. On the basis of the obtained results of own research and literature data, it was possible to determine the general model of oxidation of the Fe40Al5Cr0.2TiB alloy at the temperature of 700 °C during 2000 h.

## Figures and Tables

**Table 1:** Chemical composition of Fe40Al5Cr0.2TiB alloy.

Element	Al	Cr	Ti	B	Fe
% mas.	24.53	5.80	0.19	0.01	rest
% at.	40.10	4.86	0.18	0.06	rest

## Data Availability

The original contributions presented in this study are included in the article. Further inquiries can be directed to the corresponding authors.
